# What You Eat Matters: Nutrient Inputs Alter the Metabolism and Neuropeptide Expression in Egyptian Cotton Leaf Worm, *Spodoptera littoralis* (Lepidoptera: Noctuidae)

**DOI:** 10.3389/fphys.2021.773688

**Published:** 2021-11-04

**Authors:** Cansu Doğan, Gözde Güney, Kardelen K. Güzel, Alp Can, Dwayne D. Hegedus, Umut Toprak

**Affiliations:** ^1^Molecular Entomology Laboratory, Department of Plant Protection, Faculty of Agriculture, Ankara University, Ankara, Turkey; ^2^Laboratory for Stem Cells and Reproductive Cell Biology, Department of Histology and Embryology, School of Medicine, Ankara University, Ankara, Turkey; ^3^Agriculture and Agri-Food Canada, Saskatoon, SK, Canada; ^4^Department of Food and Bioproduct Sciences, College of Agriculture and Bioresources, University of Saskatchewan, Saskatoon, SK, Canada

**Keywords:** high-fat, high-sugar, calcium, lipid, trehalose, AKH, insulin, sNPF

## Abstract

Lipids and carbohydrates are the two primary energy sources for both animals and insects. Energy homeostasis is under strict control by the neuroendocrine system, and disruption of energy homeostasis leads to the development of various disorders, such as obesity, diabetes, fatty liver syndrome, and cardiac dysfunction. One critical factor in this respect is feeding habits and diet composition. Insects are good models to study the physiological and biochemical background of the effect of diet on energy homeostasis and related disorders; however, most studies are based on a single model species, *Drosophila melanogaster*. In the current study, we examined the effects of four different diets, high fat (HFD), high sugar (HSD), calcium-rich (CRD), and a plant-based (PBD) on energy homeostasis in younger (third instar) and older (fifth instar) larvae of the Egyptian cotton leafworm, *Spodoptera littoralis* (Lepidoptera: Noctuidae) in comparison to a regular artificial bean diet. Both HSD and HFD led to weight gain, while CRD had the opposite effect and PBD had no effect in fifth instar larvae and pupae. The pattern was the same for HSD and CRD in third instar larvae while a reduction in weight was detected with HFD and PBD. Larval development was shortest with the HSD, while HFD, CRD, and PBD led to retardation compared to the control. Triglyceride (TG) levels were higher with HFD, HSD, and PBD, with larger lipid droplet sizes, while CRD led to a reduction of TG levels and lipid droplet size. Trehalose levels were highest with HSD, while CRD led to a reduction at third instar larvae, and HFD and PBD had no effect. Fifth instar larvae had similar levels of trehalose with all diets. There was no difference in the expression of the genes encoding neuropeptides *SpoliAKH* and *SpoliILP1-2* with different diets in third instar larvae, while all three genes were expressed primarily with HSD, and *SpolisNPF* was primarily expressed with HFD in fifth instar larvae. In summary, different diet treatments alter the development of insects, and energy and metabolic pathways through the regulation of peptide hormones.

## Graphical Abstract

The parameters examined in *Spodoptera littoralis* larvae in response to four different diets, high-fat, high-sugar, calcium-rich and plant-based are shown starting with the developmental duration, weight, triglyceride and trehalose levels, lipid droplet structure, and finally with expression of four neuropeptide genes encoding adipokinetic hormone (AKH), insulin-like peptide 1 (ILP1), insulin-like peptide 2 (ILP2) and short neuropeptide F (sNPF).

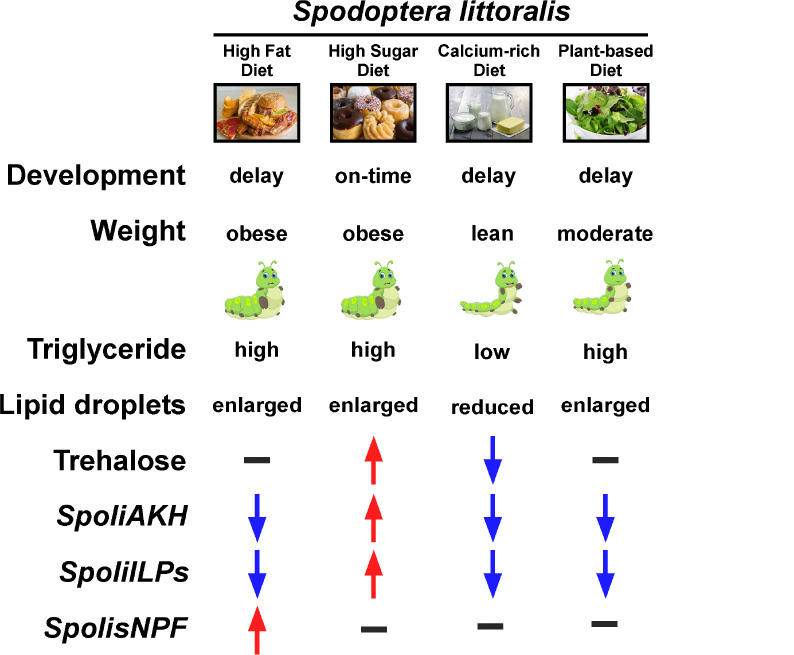

## Introduction

Energy homeostasis is essential to life, and lipids and carbohydrates are the primary energy sources for insects and animals. Maintenance of lipid and carbohydrate metabolism is critical for energy balance as an imbalance in the type and values of nutrients consumed can lead to the development of metabolic diseases and/or disorders such as obesity, diabetes mellitus, fatty liver syndrome, and cardiac dysfunction (Chatterjee and Perrimon, 2021). The commonly used model organisms in energy metabolism and nutritional studies are mice and rats. However, the fruit fly, *Drosophila melanogaster* (Diptera: Drosophilidae), has become a versatile model organism for such studies in the last decade (Álvarez-Rendón et al., 2018; Staats et al., 2018). Drosophila, like many other insects, is easy to produce in large numbers with low cost, suitable for genetic modification and large-scale screens, capable of accumulating fats in a short period of time, allowing rapid observation of metabolic changes over its short lifespan. It can also be manipulated for different phenotypes (obese, lean, Type I or Type II diabetes, etc.) by artificial diets with different fat or carbohydrate levels (Baker and Thummel, 2007; Buch et al., 2008; Birse et al., 2010; Musselman et al., 2011; Heinrichsen and Haddad, 2012; Na et al., 2013; Liao et al., 2021). More importantly, many of the disease-associated genes are also conserved in Drosophila (Reiter et al., 2001; Musselman and Kühnlein, 2018; Chatterjee and Perrimon, 2021). Studies on Drosophila demonstrated the overall potential of insects for nutrient-related metabolism studies.

Most of the studies on insect metabolism/diet interaction have focused on the outcomes of a high fat diet (HFD) or high sugar diet (HSD). Artificial diets with excessive coconut, soybean, or palm oil representing HFD, or sucrose or glucose representing HSD were used to establish diet-induced diabetes and obesity in Drosophila (Piper and Bartke, 2008; Birse et al., 2010; Musselman et al., 2011; Heinrichsen et al., 2014; Woodcock et al., 2015). The excess energy from these diets is stored as triglycerides (TGs) and glycogen in cytoplasmic lipid droplets (LDs) of the fat body, an analogous tissue to mammalian adipose tissue and the liver (Toprak et al., 2014; Güney et al., 2021). Fat body lipid and carbohydrate metabolism are under the control of hormones produced by neurosecretory cells and endocrine glands such as the corpora cardiaca (CC) and the *corpora allata* (Toprak, 2020). Understanding the operation of the fat body in response to different diets requires examination of the neuropeptides involved in lipid and carbohydrate metabolism.

Similar to that in mammals, two neuropeptides that serve as the key regulators of energy metabolism are glucagon-like adipokinetic hormone (AKH) and insulin-like peptides (ILPs) (Musselman and Kühnlein, 2018; Toprak, 2020). AKH is primarily involved in the initiation of lipid and carbohydrate mobilization from the fat body during periods of energy demand, leading to lipid hydrolysis, and is considered as a “hunger hormone” (Lee and Park, 2004; Owusu-Ansah and Perrimon, 2014; Huang et al., 2020). In contrast, central ILPs induce accumulation of TG and glycogen, larval growth, and development while reducing the insect’s primary blood sugar, trehalose, and glucose, and are considered to be “satiety hormones” (DiAngelo and Birnbaum, 2009; Owusu-Ansah and Perrimon, 2014). A third neuropeptide that induces food consumption and the secretion of ILPs from insulin-producing cells (IPCs) is the short neuropeptide F (sNPF), the insect functional homolog of mammalian Neuropeptide Y (Wu et al., 2003; Lee et al., 2004). The sNPF leads to an increase in overall body size and lipogenesis (Lee et al., 2004; Nagata et al., 2011; Baumbach et al., 2014a). Nevertheless, the links between the composition of the diet and the regulation of neuropeptides are still unclear, and determining the transcriptional response of genes encoding neuropeptides to nutrients will be critical to our understanding of this topic.

A review of the studies of dietary effects on insect energy metabolism contains several gaps. Firstly, the vast majority of the data comes from Drosophila, and little is known on the metabolism of plant pests in response to diet. Examination of diet-insect energy metabolism in phytophagous pest insects shows potential as such insects feed on plant tissues and juices that are rich in carbohydrates and successfully coordinate their energy homeostasis. Examination of the metabolism of such phytophagous insects might, therefore, provide information useful in mammalian systems. Secondly, most of these studies focused on a single diet (mostly HFD or HSD) and did not compare diets. In addition, studies with alternative diets [vegetable or with other supplements, such as calcium (Ca^2+^)] have not been examined. Thirdly, the number of studies focusing on hormonal control of energy metabolism in response to diet is limited. Finally, most of the studies were designed with short exposure to diet treatments (1–7 days) and did not examine the outcome of longer exposure to diet treatments.

The current study was aimed at understanding the effects of different diets on energy metabolism according to multiple biological and physiological parameters. Our model was a phytophagous insect species, the Egyptian cotton leafworm, *Spodoptera littoralis* (Lepidoptera: Noctuidae), a major pest of cotton and many vegetables in Europe, the Middle-East, and North Africa, particularly, around the Mediterranean basin (Hosny et al., 1986; Toprak et al., 2006). HFD, HSD, a calcium-rich diet (CRD), and a plant-based diet (PBD) were supplied throughout the larval development and compared to a standard artificial bean diet. The CRD was included as studies have indicated the involvement of Ca^2+^ signaling molecules, cellular, and dietary Ca^2+^ in lipid and carbohydrate metabolism, body weight regulation, obesity, and diabetes in insects and mammals (Zemel et al., 2000; Shi et al., 2001; Zemel, 2002; Jacqmain et al., 2003; Baumbach et al., 2014a,b; Arruda and Hotamisligil, 2015; Maus et al., 2017; Alomaim et al., 2019; Doğan et al., 2021a,b; Toprak et al., 2021). The PBD (lettuce leaves) was used as lettuce is a natural host for *S*. *littoralis* (Toprak et al., 2006) and vegetarian diets are recognized as healthy diets due to their positive effects on metabolism. The parameters examined included body weights, TG and trehalose levels, and lipid droplet size in younger (third instar) and older larvae (fifth instars). Larval developmental durations from neonate toward pupal stage were also investigated. Finally, the expression of genes encoding the AKH, ILP1, ILP2, and sNPF with each diet was examined.

## Materials and Methods

### Insects

*Spodoptera littoralis* larvae (Toprak et al., 2006) were reared at 25 ± 1°C, 60 ± 5% relative humidity under a 16:8 h (light: dark conditions) regime at Ankara University Faculty of Agriculture, Department of Plant Protection. The progenitors of the insects used in the experiments were fed an artificial bean diet (described below) until adulthood, and the adults were fed with cotton soaked with honey:water (1:10) mixture.

### Diet Treatments

*Spodoptera littoralis* neonates hatched from eggs were fed with five different diets: regular artificial bean diet (control), high sugar diet (HSD), high fat diet (HFD), calcium-rich diet (CRD), and a plant-based diet (PBD) until pupation. The artificial bean diet contained the following ingredients: 4 g ascorbic acid, 1.25 g sorbic acid, 2.5 g methyl 4-hydroxybenzoate, 3 g cereal germ, 266.5 g bean, 14 g agar, 35 g yeast extract, and 800 ml water. The HSD, HFD, and CRD included 40 g sucrose (5%), 40 ml soybean oil (5%), and 8 g CaCO3 (1%) in the artificial bean diet above, respectively. The PBD consisted of fresh lettuce leaves.

### Body Weight and Developmental Rate

The effects of diets on *S. littoralis* development and body weight were evaluated. A group of 90 neonates hatched from eggs was fed continuously on each diet (HSD, HFD, CRD, PBD, and control) under the rearing conditions described above. Upon hatching, development (from the neonate toward the pupal stage) was monitored daily. Larval instars were distinguished according to the head capsule slippage under a stereo-microscope and physical observations based on shedding of the old cuticle (EPPO, 2015). Twenty-third and fifth instar larvae and pupal stages for each diet group were randomly within the first 12 h after molting or pupation to measure weight on a high precision digital scale (ISOLAB GmbH, Germany). Experiments were replicated three times.

### Triglyceride and Trehalose Measurement Analyses

The levels of TG and the insect blood sugar, trehalose, were measured as key energetic indicators to determine the effects of different diets on *S. littoralis* larval metabolism. Triglyceride content was determined using the Triglyceride Quantification Kit according to the instructions of the manufacturer (Sigma, Germany). A pool of 10 third and 3 fifth instar larvae was flash-frozen with liquid nitrogen, ground in a mortar, and prepared for the fluorometric measurement. Trehalose content was determined using the Megazyme Trehalose Assay Kit (Megazyme, Ireland) according to the instructions of the manufacturer. A pool of 10 third and 3 fifth instar larvae was used. Absorbances at 590 and 340 nm were measured for TG and trehalose, respectively, using a SpectraMax M2 Microplate Reader (Molecular Devices, United States). All experiments were replicated three times.

### Microscopy

To visualize the lipid droplets, the fat body from five third and five fifth instar larvae from each diet group were dissected in Ringer’s solution (153 mM NaCl, 2.68 mM KCl, 1.36 mM CaCl_2_⋅2H_2_O). The tissues were fixed overnight in 10% formaldehyde. Samples were stained with 1 μg/ml Nile red dye (Cat No: 72485, Sigma-Aldrich), washed two times with ddH_2_O, and treated with Hoechst dye (1 μg/ml in 1:1 PBS/glycerol), and covered with a coverslip. A confocal microscope (Zeiss LSM 880 fast airscan confocal system, Germany) was used to visualize the lipid droplet in the fat body tissue. Nile red signals were scanned at 543 nm at a 1 μm interval to construct a 25 μm image stack.

### Lipid Droplet Size

Three slides were randomly selected from each fat body tissue sample from each diet for lipid droplet size measurement using the area measurement tool (ZEN 3.3, blue edition). Each image was scaled to 20 μm, and the lipid droplets were marked manually using freehand selection using the measure tool from the length section. All lipid droplets on the imaged slides were counted except the lipid droplets at the edges of the image that were partially cut off.

### Reverse-Transcription Quantitative PCR

RT-qPCR analyses were conducted to examine the effect of diet on the expression of four neuropeptide genes, namely, *SpoliAKH* (cDNA sequence provided by Dr. Heiko Vogel, Max Planck Institute for Chemical Ecology, Germany), *SpoliILP1* (HQ451072), *SpoliILP2* (HQ451073) (Van de Velde et al., 2007), and *SpolisNPF* (AFW19647). Seven-third and three-fifth instar larvae were selected from each diet group and the control group and flash-frozen with liquid nitrogen. After grinding in a mortar, total RNA was isolated using the PureLink^TM^ RNA Mini Kit (Invitrogen) according to the instructions of the manufacturer. cDNA was synthesized from RNA using the iScript^TM^ cDNA Synthesis Kit (Bio-Rad Laboratories Inc.) according to the instructions of the manufacturer and quantified using a CFX96 Touch^TM^ Real-Time PCR Detection System (Bio-Rad Laboratories) and an SYBR Green qPCR kit (Bio-Rad Laboratories Inc.). Primers developed by Gautam et al. (2020) were used for expression analysis of *SpoliAKH*, while primers to amplify transcripts from *SpoliILP1*, *SpoliILP2*, and *SpolisNPF* were designed using Primer3Plus software with the following parameters: product size = 70–180 bp, Tm = 59–61°C, GC% = 40–60%, Max Poly-base = 3 (Table 1). *Actin* was used as the endogenous reference gene for normalizing gene transcript abundance (Večeřa et al., 2012). Data were obtained from three biological replicates and presented as relative mRNA transcript abundance (means of measurements ± SE).

**TABLE 1:** Real-time primers used in the study.

**Primer**	**Sequence**	**Source**
AKH Fp	5′-TGCGCAGATCACGTTCAG-3′	Gautom et al. 2020
AKH Rp	5′-CGACACAGCCTGGTGAACT-3′	Gautom et al. 2020
ILP1 Fp	5′-ACAGCAACATCGGACCAAGT-3′	Current study
ILP1 Rp	5′-GGACGGAACCTGGCTATCAC-3′	Current study
ILP2 Fp	5′-GGGCGTCGAGGATCCTTAAG-3′	Current study
ILP2 Rp	5′-AAGGATGACGTCGGTAGTGC-3′	Current study
sNPF Fp	5′-TGCCTCCTCAAGCTCCTTTG-3′	Current study
sNPF Rp	5′-GGTACAGCGCGTTCTTCAGA-3′	Current study
Actin Fp	5′-CGAGCGAGAAATCGTGCGTAA-3′	Večeřa et al., 2012
Actin Rp	5′-TGACTTGTCCGTGGGGAAGTT-3′	Večeřa et al., 2012

### Data Analysis

Real-time data analyses were performed using CFX Maestro^TM^ Software. Statistical significances within each sample set were determined by using one-way ANOVA, followed by a Tukey HSD test, while a *t*-test was used for binomial comparisons (diet treatment vs control). *p*-values less than.05 were considered to be significant in all.

## Results

### Development and Growth of *Spodoptera littoralis* on Different Diets

**FIGURE 1:**
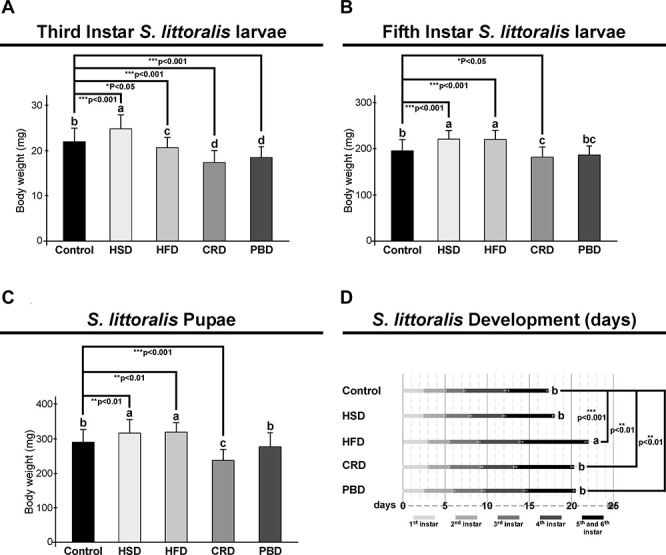
Effects of different diet treatments on the larval and pupal weights, and development of *Spodoptera littoralis*. Body weights at third **(A)**, fifth instar **(B)** larvae, and pupa **(C)**, and larval development durations **(D)** are shown. Statistical significances in the entire sample set were determined by using one-way ANOVA, followed by a Tukey HSD test (groups associated with different letters are significantly different from each other, *P* < 0.05). A *t*-test was used for binomial comparisons (diet treatment vs control) (*p < 0.05, **p < 0.01, and ***p < 0.001 indicate the statistical differences between the particular diet and the control). Values are expressed as means ± standard error. Control, Regular artificial bean diet; HSD, High sugar diet; HFD, High fat diet; CRD, Calcium-rich diet; PBD, Plant-based diet.

In third instar larvae, the highest larval body weight was detected with HSD, followed by the control diet, and then HFD, while the lowest larval body weight was detected at CRD and PBD (*p* < 0.05, Figure 1A). Larval weights for each diet treatment were statistically different in comparison to the control diet (*p* < 0.05 or *p* < 0.001) (Figure 1A). The weight of HSD-fed larvae was higher than that of the control diet (*p* < 0.001), while HFD (*p* < 0.05), CRD (*p* < 0.001), and PBD-fed (*p* < 0.001) larvae weighted less compared to that of control (Figure 1A). In fifth instar larvae, the highest body weight was detected with HSD and HFD, followed by the control diet and PBD, while the lowest larval body weight was detected with CRD (*p* < 0.05, Figure 1B), though the difference between PBD-CRD was not significant. The weights from the fifth instar larvae fed with HSD, HFD, or CRD were statistically different than those fed the control diet (*p* < 0.05 or *p* < 0.001) (Figure 1B). The weights of HSD and HFD-fed larvae were higher than that of the control diet (*p* < 0.001), while the weight of CRD-fed larvae (*p* < 0.05) was lower in comparison to the control (Figure 1B). Pupal weights were highest with HSD and HFD, followed by the control diet and PBD, while the lowest pupal weight was with CRD (*p* < 0.05, Figure 1C). The weights of the pupa from HSD, HFD, or CRD were statistically different in comparison to the control diet (*p* < 0.01 or *p* < 0.001) (Figure 1C). The weights of the pupa from HSD and HFD groups were higher than that of the control diet (*p* < 0.01), while those from the CRD group (*p* < 0.001) weighted less in comparison to control (Figure 1C).

Development periods from the neonatal stage to the pupal stage were 17.3, 18, 22, 20.3, and 20.5 days at control, HSD, HFD, CRD, and PBD, respectively (Figure 1D). The longest larval development duration was obtained at HFD; however, the differences in the developmental durations with the other diets were statistically insignificant (*p* < 0.05, Figure 1D). Development periods for the larvae fed with HFD, CRD, and PBD were statistically different than those of larvae fed with the control diet (*p* < 0.001 or *p* < 0.01) (Figure 1D). HFD, CRD, and PBD had to 4.7, 3.0, and 3.2 days of larval development delay, respectively, in comparison to control, while the.7 day of delay from HSD-exposed larvae was found insignificant compared to the control diet (*p* > 0.05).

### Lipid and Carbohydrate Metabolism of *Spodoptera littoralis* Larvae Fed With Different Diets

**FIGURE 2:**
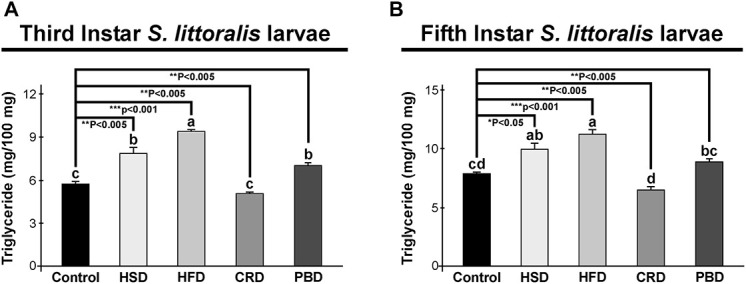
Effects of different diet treatments on the triglyceride levels of *Spodoptera littoralis*. Triglyceride levels at third **(A)** and fifth instar **(B)** larval bodies are shown as mg in 100 mg body weight. Statistical significances in the entire sample set were determined by using one-way ANOVA, followed by a Tukey HSD test (groups associated with different letters are significantly different among each other, *p* < 0.05). A *t*-test was used for binomial comparisons (diet treatment vs control) (*p < 0.05, **p < 0.005, ***p < 0.001 indicate the statistical differences between the particular diet and the control). Values are expressed as means ± standard error. Control, Regular artificial bean diet; HSD, High sugar diet; HFD, High fat diet; CRD, Calcium-rich diet; PBD, Plant-based diet.

Triglyceride and trehalose contents were measured in third and fifth instar larvae reared on different diets. In third instar larvae, the TG content was highest in larvae fed with HFD (9.4 mg/100 mg sample), followed by HSD (7.9 mg/100 mg sample), and PBD (7 mg/100 mg sample) at similar levels, and lowest with the control diet (5.8 mg/100 mg sample) and CRD (5.1 mg/100 mg sample) (*p* < 0.05) (Figure 2A). Triglyceride levels from the third instar larvae fed each diet were statistically different than those fed the control diet (*p* < 0.005) (Figure 2A). The third instar fed with HFD, HSD, and PBD had increased TG levels, while those fed CRD had reduced TG levels in comparison to the control diet (*p* < 0.005). In fifth instar larvae, the highest TG content was detected at similar levels with HFD (11.3 mg/100 mg sample) and HSD (9.9 mg/100 mg sample), followed by PBD (8.9 mg/100 mg sample) and the control diet (7.9 mg/100 mg sample), and was lowest with CRD (6.5 mg/100 mg sample) (*p* < 0.05) (Figure 2B). Notably, the difference between HSD and PBD in fifth instar larvae was not statistically significant (*p* > 0.05) (Figure 2B). Triglyceride levels from fifth instar larvae fed each diet were statistically different than those fed the control diet (*p* < 0.05) (Figure 2B). HFD, HSD, and PBD led to increased TG levels, while CRD led to reduced TG levels in fifth instar larvae in comparison with the control diet (*p* < 0.05).

**FIGURE 3:**
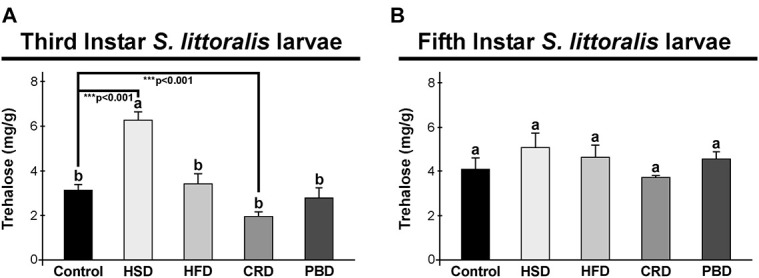
Effects of different diet treatments on the trehalose levels of *Spodoptera littoralis*. Trehalose levels at third **(A)** and fifth instar **(B)** larval bodies are shown as mg in 1 g body weight. Statistical significances in the entire sample set were determined by using one-way ANOVA, followed by a Tukey HSD test (groups associated with different letters are significantly different among each other, *p* < 0.05). A *t*-test was used for binomial comparisons (diet treatment vs control) (***p < 0.001 indicate the statistical differences between the particular diet and the control). Values are expressed as means ± standard error. Control, Regular artificial bean diet; HSD, High sugar diet; HFD, High fat diet; CRD, Calcium-rich diet; PBD, Plant-based diet.

In third instar larvae, the highest trehalose content was detected with the HSD group (6.3 mg/g sample) (*p* < 0.05) (Figure 3A), while all other diets led to similar trehalose levels (1.9–3.4 mg/g sample) (*p* > 0.05) (Figure 3A). Trehalose levels in the third instar larvae fed with HSD and CRD were statistically different from those fed with the control diet (*p* < 0.001) (Figure 3A). Larvae fed with HSD had twice (99%) trehalose levels when compared to those fed with the control diet while feeding on CRD led to a 61% reduction in trehalose levels in comparison to the control diet group (*p* < 0.001) (Figure 3A). Interestingly, in fifth instar larvae, trehalose levels did not differ significantly with any diet (*p* > 0.05) (3.8–5.1 mg/g sample) (Figure 3B).

### Microscopic Investigation of Fat Body of *Spodoptera littoralis* Fed With Different Diets

**FIGURE 4:**
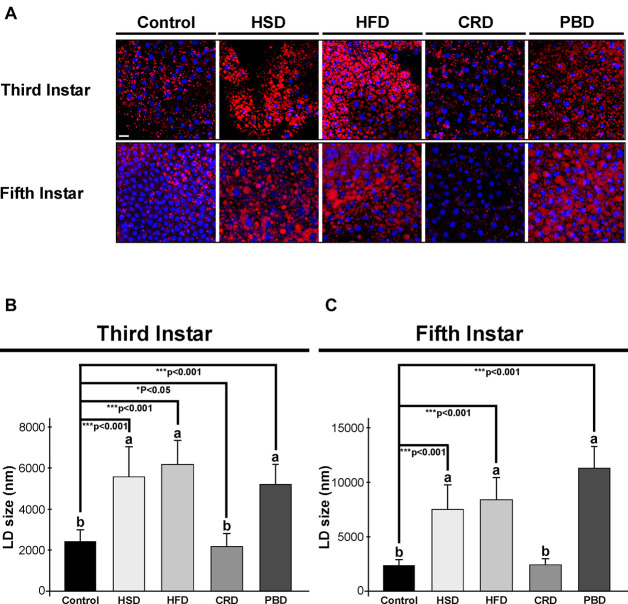
Effects of different diet treatments on the fat body lipid droplets of *Spodoptera littoralis*. The depict lipid droplets using confocal microscopy in fat body tissue from third or fifth instar larvae **(A)**, and lipid droplet sizes at third **(B)** and fifth instar larvae **(C)** are shown. Lipid droplets stained with Nile red dye in red, nuclei stained with Hoechst in blue. Statistical significances in the entire sample set were determined by using one-way ANOVA, followed by a Tukey HSD test (groups associated with different letters are significantly different among each other, *p* < 0.05). A *t*-test was used for binomial comparisons (diet treatment vs control) (*p < 0.05, ***p < 0.001 indicate the statistical differences between the particular diet and the control). Values are expressed as means ± standard error. Control, Regular artificial bean diet; HSD, High sugar diet; HFD, High fat diet; CRD, Calcium-rich diet; PBD, Plant-based diet. Scale bar, 20 μm in panel **(A)**.

Lipid droplets stained with Nile red dye (red) and nuclei stained with Hoechst (blue) from the fat body of third and fifth instar larvae fed with different diets are illustrated in Figure 4A. In accordance with TG measurements, the highest number of lipid droplets were detected in both third and fifth instar larvae fed with HFD and HSD (Figure 4A). Lipid droplets were also visible at high amounts in both instars fed with PBD. There was an obvious decrease in the amount of lipid droplets in the larvae fed with CRD (Figure 4A).

The sizes of the lipid droplets from the fat body of larvae fed with different diets were also determined. Both larval stages exhibited a similar pattern of lipid droplet size in response to diet (Figures 4B,C). In third instar larvae, the average sizes of the lipid droplets were 2,524, 5,739, 6,354, 2,240, and 5,360 nm in the control, HSD, HFD, CRD, and PBD groups, respectively, while the corresponding values were 2,359, 7,578, 8,394, 2,446, and 11,299 nm with fifth instar larvae. Larvae fed with HFD, HSD, and PBD had the highest lipid droplet size, while the CRD and control group had lower lipid droplet sizes (*p* < 0.05). Feeding with HFD, HSD, and PBD led to increased lipid droplet size (*p* < 0.001) while feeding with CRD led to reduced lipid droplet size (*p* < 0.05) in comparison to the control diet in third instar larvae (Figure 4B). Similarly, lipid droplets from the fifth instar larvae fed with PBD, HFD, and HSD were also statistically different than those fed the control diet (*p* < 0.001) (Figure 4C). Feeding with PBD, HFD, and HSD led to increased lipid droplet size (*p* < 0.001), while the difference between the droplet sizes in larvae fed with CRD or the control diet was insignificant in fifth instar larvae (*p* > 0.05) (Figure 4B).

The size of the lipid droplets in larvae fed with HSD, HFD, and PBD significantly increased from the third instar to fifth instar 1. 3-, 1. 3-, and 2-fold, respectively (*p* < 0.001) (Figures 4B,C). It is also noteworthy that the lipid droplets from third instar larvae fed with PBD were both smaller and larger in size, while those from fifth instar larvae were all large and uniform in size.

### Expression of Neuropeptides of *Spodoptera littoralis* Fed With Different Diets

**FIGURE 5:**
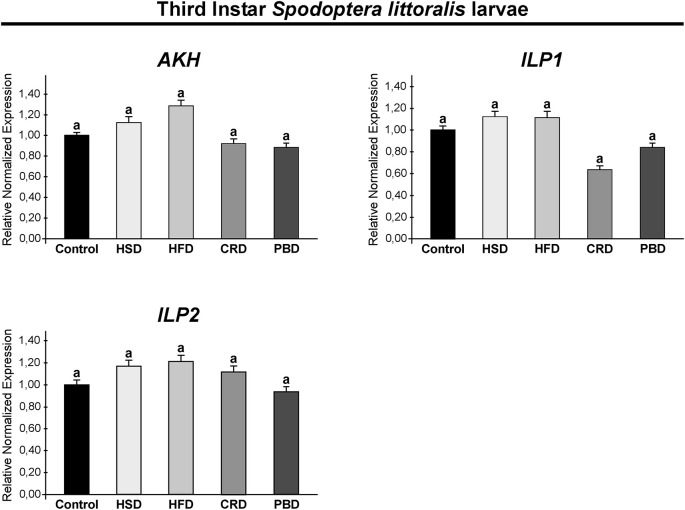
Effects of different diet treatments on the expression of *Spodoptera littoralis* adipokinetic hormone (*SpoliAKH*), insulin-like peptide 1 (*SpoliILP1*), and insulin-like peptide 2 (*SpoliILP2*) at third instar larvae by Real-time PCR. *Spodoptera littoralis* actin gene was used as the internal reference. The statistical significance of transcript abundance differences among groups was determined through ANOVA, followed by the Tukey HSD test (groups associated with different letters are significantly different from each other, *p* < 0.05). Values are expressed as means ± standard error. Control, Regular artificial bean diet; HSD, High sugar diet; HFD, High fat diet; CRD, Calcium-rich diet; PBD, Plant-based diet.

**FIGURE 6:**
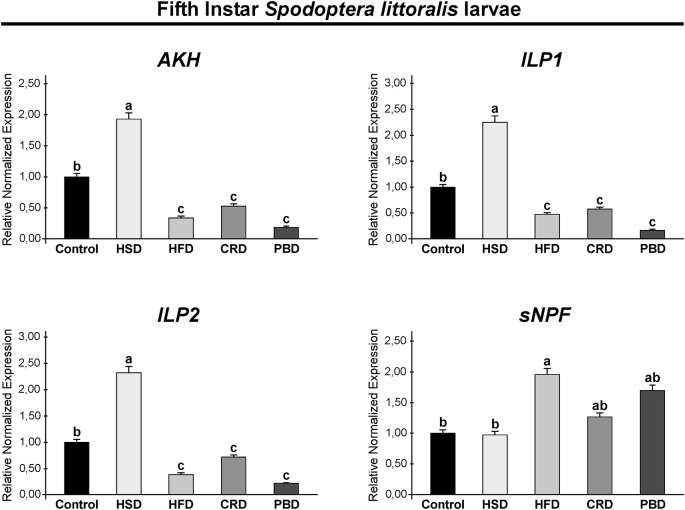
Effects of different diet treatments on the expression of *Spodoptera littoralis* adipokinetic hormone (*SpoliAKH*), insulin-like peptide 1 (*SpoliILP1*), insulin-like peptide 2 (*SpoliILP2*), and short neuropeptide F (*SpolisNPF*) at fifth instar larvae by Real-time PCR. *Spodoptera littoralis* actin gene was used as the internal reference. The statistical significance of transcript abundance differences among groups was determined through ANOVA, followed by the Tukey HSD test (groups associated with different letters are significantly different from each other, *p* < 0.05). Values are expressed as means ± standard error. Control, Regular artificial bean diet; HSD, High sugar diet; HFD, High fat diet; CRD, Calcium-rich diet; PBD, Plant-based diet.

Expression of *SpoliAKH*, *SpoliILP1*, *SpoliILP2*, and *SpolisNPF* was examined in third and fifth instar larvae in response to different diet treatments. Different diet treatments did not alter the transcript abundance of *SpoliAKH*, *SpoliILP1*, and *SpoliILP2* in the third stage of larvae (*p* > 0.05) (Figure 5). The *SpolisNPF* mRNA was undetectable in the third stage of larvae [quantification cycle (Cq) value ≥ 40]. On the other hand, different diet treatments led to significant changes in the expression of these genes in fifth instar larvae (Figure 6). *SpoliAKH*, *SpoliILP1*, and *SpoliILP2* were primarily expressed in larvae fed with HSD while expression of these genes in larvae fed with HFD, CRD, and PBD was similar but lower than that of control (*p* < 0.05) (Figure 6). Transcript abundance of *SpolisNPF* was significantly higher in larvae fed with HFD. However, HSD, CRD, and PBD led to similar levels of expression with control diet (*p* < 0.05) (Figure 6).

## Discussion

### Growth and Development of *Spodoptera littoralis* Fed With Different Diets

Our study revealed that different diet treatments alter insect growth and development. Unsurprisingly, the highest larval and pupal weight gains were obtained with HSD and/or HFD, except for third instar larvae. Similarly, HFD led to a significant increase in the bodyweight of adult flies (Trindade de Paula et al., 2016) and pupa (Reed et al., 2010). In contrast, HFD led to smaller sizes and decreased growth rates in the lepidopteran, *Manduca sexta* (Cambron et al., 2019). A similar decrease in weight or body size was reported for third instar Drosophila larvae (Musselman et al., 2011) and adults (Schultzhaus et al., 2018) in response to HFD. Such a decrease in response to HFD was detected only in third instar *S*. *littoralis* larvae here. Cambron et al. (2019) suggested that caterpillars exposed to HFD eat less and might feel “sick” due to consuming high amounts of lipids in the diet, resulting in smaller individuals. Similar to our results on HSD, increased body weight has been reported in larvae and pupa of the predator *Harmonia axyridis* (Coleoptera: Coccinellidae) (Li et al., 2020), adult Drosophila (Morris et al., 2012; Rovenko et al., 2015a), and the mosquito *Ae. aegypti* (van Schoor et al., 2020). In contrast, weight or body size decrease has been reported in Drosophila and *Ae. aegypti* larvae or adults exposed to HSD (Musselman et al., 2011, 2019; Pasco and Léopold, 2012; van Schoor et al., 2020). On the other hand, CRD led to lower weights in all stages in comparison to the control here. There has not been a comparative study on insects on this topic, to the best of our knowledge. However, studies with mammals indicate that dietary Ca^2+^ might lead to weight loss (Zemel et al., 2000; Shi et al., 2001; Zemel, 2002; Jacqmain et al., 2003; Sun and Zemel, 2004). In the present study, PBD also led to lower (third instar larvae) or similar weights (fifth instar larvae or pupa); However, different PBDs might lead to different outcomes. The contradictory results on body weight, size, or mass-diet interaction in the literature are likely to be related to multiple factors, such as the source and percentage of fats (Fernando-Warnakulasuriya et al., 1988; Trindade de Paula et al., 2016; Cambron et al., 2019; Liao et al., 2021) or carbohydrates (Musselman et al., 2011, 2019) used in the diets.

Larval developmental durations from the neonatal to the pupal stage with different diets were also assessed. All diets, except HSD, led to a delay in development. This was most dramatic with HFD (a delay of 4.7 days), while CRD and PBD led to delays of 3 and 3.2 days, respectively. Similarly, an HFD was found to cause a developmental delay in the phytophagous caterpillar *M. sexta* (Fernando-Warnakulasuriya et al., 1988). Such developmental delays might be related to decreased food consumption, which was reported for *M. sexta* larvae in response to HFD (Cambron et al., 2019). However, HFD led to increased weight gain in fifth instar larvae despite a significant delay in larval development. This suggests that decreased food consumption may not be the reason for the coincidental developmental delay. On the other hand, HSDs have been reported to accelerate larval development in Drosophila (Tatum and Beadle, 1939), *H. axyridis* (Li et al., 2020), and *Ae*. *aegypti* (van Schoor et al., 2020) while developmental delays in response to HSD with excessive levels of carbohydrates have been reported (Rovenko et al., 2015a,b; van Schoor et al., 2020). Relatively recent studies on Drosophila found that HSD reduces the sensitivity of the brain to sweet flavors, leading to over-eating and weight gain (May et al., 2019, 2020). Here, as HSD led to increased weight gain without any developmental delay, such induction of feeding in *S*. *littoralis* is likely to occur in response to HSD. On the other hand, CRD led to lower weight gains with a delay in larval development, suggesting *S*. *littoralis* larvae are likely feeding less on this diet. The PBD also led to lower weight gain in third instar larvae, while the weights of fifth instar larvae and pupa were comparable to the control diet group. However, it is not clear to us why larvae exhibited a developmental delay on a natural plant diet, in this case, lettuce.

### Energy Processes of *Spodoptera littoralis* Larvae Fed With Different Diets

In the current study, the highest TG levels were obtained with HFD and HSD. In the third instar larvae, TG levels increased around 63 and 37% with HFD and HSD, respectively, while the respective increases were 42 and 26% in fifth instar larvae. This suggests that there is a greater increase in TG levels with HFD compared to HSD, and with third instar larvae compared with fifth instar larvae. The literature on the effect of HFD on lipid levels is similar to our results. *Manduca sexta* larvae reared on HFD were found to possess higher lipid content in their fat body (Cambron et al., 2019). Various studies from *D. melanogaster* also revealed that HFD leads to increased TG levels in adults (Birse et al., 2010; Heinrichsen and Haddad, 2012; Heinrichsen et al., 2014; Woodcock et al., 2015; Trindade de Paula et al., 2016; Jung et al., 2018; Rivera et al., 2019; Liao et al., 2021). The HFD-induced TG accumulation observed here and by others is not surprising as excess dietary fatty acids are converted to TG for storage in fat body lipid droplets (Carvalho et al., 2012; Toprak et al., 2020). As seen with third instar larvae here, HFD-induced TG increase does not necessarily have to lead to an increase in weight or size as was reported for *M*. *sexta* larvae (Cambron et al., 2019) or *D. simulans* flies (Murashov et al., 2020). On the other hand, HSD-induced TG levels have been reported in Drosophila adults (Na et al., 2013; Rovenko et al., 2015a; May et al., 2019; Rani et al., 2020) and larvae (Musselman et al., 2011, 2019; Pasco and Léopold, 2012) and in *Ae. aegypti* adults (van Schoor et al., 2020). Interestingly, PBD also led to a TG increase of around 22% in the third instar while a lower level of increase (12%) was detected in the fifth instar. Carvalho et al. (2012) reported that carbohydrates in a PBD led to an accumulation of TG in the fat body of Drosophila larvae, and the genes involved in fat biosynthesis were upregulated in response to excess dietary carbohydrates (Zinke et al., 2002; Musselman et al., 2011). In the black soldier fly, *Hermetia illucens*, a vegetable-mixed diet also led to higher amounts of lipids (Pimentel et al., 2017). The HSD or PBD-induced TG accumulation is not surprising as acetyl-CoA derived from the metabolism of dietary carbohydrates is converted into fatty acids and stored as TGs in the fat body, which is considered as a protective mechanism against glucotoxicity (Musselman et al., 2013; Palanker Musselman et al., 2016). A more exciting finding in the current study is that the CRD led to decreased TG content. Specifically, the decreases were around 14 and 12% in third and fifth instar larvae, respectively. Various studies in mammals and insects revealed the involvement of dietary Ca^2+^ and specific Ca^2+^ signaling genes in lipid metabolism, leading to lipolysis (Allen and Beck, 1986; Shi et al., 2001; Zemel, 2002; Jacqmain et al., 2003; Arruda and Hotamisligil, 2015; Maus et al., 2017; Doğan et al., 2021a,b; Toprak et al., 2021). In Drosophila, elevated cytosolic Ca^2+^ levels lead to activation of lipolysis (Subramanian et al., 2013a,b; Baumbach et al., 2014a,b; Bi et al., 2014).

Trehalose, the non-reducing disaccharide of glucose, serves as the primary sugar circulating in the hemolymph of most insects and plays an essential role in systemic energy homeostasis (Ugrankar et al., 2015; Yasugi et al., 2017). The dietary sugar-linked synthesis of trehalose in the fat body is critical in balancing the fluctuation of blood sugar levels (Yasugi et al., 2017). In the current study, the highest trehalose level was detected in third instar larvae exposed to HSD. The HSD led to almost double the trehalose levels, while CRD led to a 61% reduction. HSD-induced trehalose increases have been reported for other insects, including the larvae of *M*. *sexta* (Thompson et al., 2003), *Helicoverpa zea* (Lepidoptera: Noctuidae) (Friedman et al., 1991), larvae and adults of *H*. *axyridis* (Li et al., 2020), nymphs of *Locusta migratoria* (Orthoptera: Acrididae) (Zanotto et al., 1996), and larvae (Musselman et al., 2011; Pasco and Léopold, 2012) or adults (Morris et al., 2012; Na et al., 2013; Rovenko et al., 2015b; Rani et al., 2020) of Drosophila. On the other hand, high amounts of trehalose in the fifth instar larvae at any diet might be related to the increased energy demand for the upcoming pupal stage. Matsuda et al. (2015) also reported a gradual decrease in trehalose levels during metamorphosis due to its high utilization during pupation. Furthermore, deficiency of trehalose-6-phosphatase, the enzyme involved in trehalose synthesis in the fat body, leads to lethality in the pupal stage, but not in the larval stage (Ugrankar et al., 2015).

A confocal microscopic investigation confirmed the higher lipid accumulation with HFD and HSD. High amounts of lipid droplets were also detected in larvae fed with PBD in accordance with the TG measurements with this diet. The lipid droplets were largest with HSD, HFD, and PBD, which had 2. 3-, 2. 5-, and 2.1-fold increases, respectively, compared to control in third instar larvae, while respective values of 3. 2-, 3. 6-, and 4.8-fold were observed in fifth instar larvae. Increased lipid droplet size has also been reported in Drosophila larvae fed with HSD (Musselman et al., 2011), adults fed with HFD (Birse et al., 2010), and in *H. illucens* larvae fed with PBD (Pimentel et al., 2017). PBD was the only diet that led to variability in lipid droplet size as third instar larvae that were fed this diet possessed both small and large lipid droplets, while those from fifth instar larvae were uniform (all large). Additionally, the size of the lipid droplets with PBD increased 2.1-fold from third to the fifth instar, while a lower level of increase (1.3-fold) was observed with HSD and HFD, suggesting a possible effect of plant ingredients on lipid droplet size. On the other hand, CRD led to a lower amount of lipids with smaller lipid droplet sizes compared to that from the high-calorie diets in accordance with the lower TG measurements detected with CRD. The decrease in lipid droplet size between CRD and the control group was around 13% in third instar larvae, while the difference in fifth instar larvae was insignificant. Furthermore, lipid droplet sizes with CRD were reduced from 2.6- to 3. 1-, and 2.8- to 3.4-fold with HSD and HFD, respectively. In summary, dietary Ca^2+^ leads to a reduction in TG levels and lipid droplet size.

### Endocrine/Neuropeptide Regulation of *Spodoptera littoralis* Fed With Different Diets

Energy processes are under the strict control of the neuroendocrine system and are primarily regulated by AKH and ILPs (Toprak, 2020). Here, we examined the expression of *SpoliAKH*, *SpoliILP1*, and *SpoliILP2* in response to feeding on different diets. In third instar larvae, no difference was detected in the expression of *SpoliAKH*, *SpoliILP1*, and *SpoliILP2* in response to any diet, while in the fifth instar larvae, all three genes had different expression profiles in response to diet.

The main role of ILPs is to lower blood sugar and to coordinate the conversion of excess sugar into TGs and glucogen in the fat body (DiAngelo and Birnbaum, 2009; Semaniuk et al., 2018). *SpoliILP1* and *SpoliILP2* revealed the same pattern with the highest expression with HSD and a lower expression with HFD, CRD, and PBD. Both genes were up-regulated in response to HSD and downregulated with HFD, CRD, and PBD in comparison to the control in fifth instar larvae. It is easier to explain the higher expression of ILPs with HSD. HSD led to the highest weight gains and TG levels in fifth instar larvae (in this study), and central ILPs induce lipogenesis (DiAngelo and Birnbaum, 2009; Semaniuk et al., 2018; Liao et al., 2021). Similarly, HSD led to increased expression of central ILP genes in Drosophila larvae (Musselman et al., 2011; Pasco and Léopold, 2012) and adults (Morris et al., 2012; Na et al., 2013; Rovenko et al., 2015a; Rani et al., 2020). On the other hand, it is not surprising to detect the downregulation of ILP genes with CRD as this diet led to lower weight gain and TG levels. Disruption of Ca^2+^ signaling was found to lead to up-regulation of *DILP2* in Drosophila (Jayakumar et al., 2016). Lower expression of ILP genes was also detected with PBD, while this diet led to similar weight gain and TG levels compared to the control. More interestingly, ILP gene expression was not affected by another high calorie diet, specifically HFD, despite the fact that this diet also led to increased weight and TG content at comparable levels to HSD in the fifth instar. Birse et al. (2010) reported downregulation of *DILP2* in Drosophila adults 5 days after feeding with HFD. Similarly, Liao et al. (2021) reported reduced DILP2 peptide levels in Drosophila adults fed with HFD despite increased levels of lipids. The absence of up-regulation of SpoliILP genes with HFD might be related to the fact that the fifth instar *S*. *littoralis* larvae have already accumulated high levels of TG directly from the diet. Therefore, they do not need to accumulate more fats.

The main role of the AKH is to initiate mobilization of fat and carbohydrate reserves in response to energy-demanding processes such as flight, fasting, and exercise similar to its mammalian ortholog, glucagon (Gäde and Auerswald, 2003; Grönke et al., 2007). Studies on Drosophila adults revealed that AKH leads to hydrolysis of stored TGs and glycogen into diglyceride and trehalose, respectively (Lee and Park, 2004; Gáliková et al., 2015). In the current study, *SpoliAKH* was highly expressed with HSD and expressed at lower levels with HFD, CRD, and PBD. Such a high expression with HSD might be related to the preparation for the increased energy demand at the onset of metamorphosis. HSD is an efficient fuel with a high and rapid energy supply. Furthermore, this diet led to the shortest developmental duration. On the other hand, AKH maintains trehalose levels (Gáliková et al., 2015), and high trehalose levels promote AKH secretion in Drosophila larvae (Kim and Neufeld, 2015). This is in accordance with the high levels of trehalose and *AKH* expression detected with HSD. Song et al. (2017) reported enhanced expression of *AKH* in the fat body in response to HSD in Drosophila. Musselman et al. (2011) also reported up-regulation of the AKH receptor gene in response to HSD in Drosophila larvae, despite up-regulation of lipogenesis and trehalose synthesis. It is noteworthy that AKH levels might be affected by ILP levels, or the AKH levels might regulate ILP secretion. DILP2 induces *AKH* expression in CC (Post et al., 2019) and AKH signaling is necessary for sugar-dependent DILP3 release (Kim and Neufeld, 2015). Therefore, the AKH/insulin interaction is not necessarily antagonistic (Buch et al., 2008). Accordingly, the expression pattern of *SpoliAKH* was similar to that of *SpoliILP1* and *SpoliILP2* in this study, suggesting they work in a complementary manner. On the other hand, the other high calorie diet, HFD, led to the downregulation of *SpoliAKH*. Several days of HFD treatment did not alter the expression or peptide levels of AKH in Drosophila adults (Huang et al., 2020). A previous study by Lee and Park (2004) also reported that disruption of AKH signaling did not have a significant effect on stored TG levels. These findings, together with ours, suggest that an HFD does not necessarily have to lead to upregulation of *AKH* transcription or an increase in peptide level. Furthermore, AKH levels do not have to be negatively correlated with TG levels (Liao et al., 2021). On the other hand, lower expression of *AKH* with CRD supports the observation that this diet leads to lower levels of weight and TG levels, which is an indication of decreased energy mobilization. Overall, *AKH* expression interferes with trehalose levels and, therefore, primarily responds to high carbohydrate diets other than diets rich in fat (Lee and Park, 2004; Grönke et al., 2007; Gáliková et al., 2015) as was also detected here.

Short neuropeptide F is a functional homolog of Neuropeptide Y, a strong orexigenic peptide that promotes diet-induced obesity in mammals (Baumbach et al., 2014a). We also examined the expression of *SpolisNPF* in response to different diets. *SpolisNPF* mRNA was not detectable in third instar larvae. *SpolisNPF* expression in fifth instar larvae was higher in the HFD group, while all other treatments (HSD, CRD, and PBD) led to similar levels of expression. Studies in Drosophila report a stimulatory effect of sNPF on feeding; however, there are also studies reporting cessation of feeding by sNPF (Lee et al., 2004; Nagata et al., 2011; Baumbach et al., 2014a; Dillen et al., 2014; Slade and Staveley, 2016). The high expression of *SpolisNPF* with HFD appears to be in accordance with the increased weight and TG levels in fifth instar *S*. *littoralis* larvae and the stimulatory role of sNPF on feeding and fat accumulation in Drosophila. Furthermore, Stobdan et al. (2019) and Huang et al. (2020) reported HFD-induced hyperphagia in Drosophila adults. On the other hand, sNPF was found to inhibit AKH secretion from the CC in Drosophila adults (Oh et al., 2019). In this manner, high expression of *SpolisNPF* with HFD, CRD, and PBD might be a reason for the downregulation of *SpoliAKH* with these diets. Notably, sNPF might relate more to regulating metabolism and growth rather than feeding (Lin et al., 2019). Therefore, the exact role of SpolisNPF needs to be elucidated in caterpillars, including *S*. *littoralis*.

## Conclusion

In conclusion, both HSD and HFD led to increased weight gain, while CRD led to a reduced weight gain, and PBD did not alter the weight gain. Larval development was shortest with HSD, while HFD led to significant retardation, and CRD and PBD led to moderate retardations. TG levels were higher with HFD, HSD, and PBD with larger lipid droplets, while CRD led to a reduction in TG levels and lipid droplet size. Trehalose levels were highest in HSD, while CRD led to a reduction in third instar larvae. There was no diet effect on trehalose levels in fifth instar larvae. No difference was detected in the expression of *SpoliAKH* and *SpoliILP1-2* between diets in third instar larvae, while all three genes were primarily expressed with HSD, and *SpolisNPF* was expressed in fifth instar larvae fed with HFD. Further investigations on peptide levels can add to our understanding of the neuroendocrine control of metabolism.

## Data Availability Statement

The original contributions presented in the study are included in the article/supplementary material, further inquiries can be directed to the corresponding author/s.

## Author Contributions

UT conceived and designed the research. CD, GG, KG, and AC performed the experiments and analyzed the data. CD, DH, and UT drafted the manuscript. DH and UT wrote the final version of the manuscript. All authors contributed to the article and approved the submitted version.

## Conflict of Interest

The authors declare that the research was conducted in the absence of any commercial or financial relationships that could be construed as a potential conflict of interest.

## Publisher’s Note

All claims expressed in this article are solely those of the authors and do not necessarily represent those of their affiliated organizations, or those of the publisher, the editors and the reviewers. Any product that may be evaluated in this article, or claim that may be made by its manufacturer, is not guaranteed or endorsed by the publisher.

## References

[B1] Allen D. O. Beck R. R. (1986). Role of calcium ion in hormone-stimulated lipolysis. *Biochem. Pharmacol.* 35 767–772. 10.1016/0006-2952(86)90244-3 3006689

[B2] Alomaim H. Griffin P. Swist E. Plouffe L. J. Vandeloo M. Demonty I. (2019). Dietary calcium affects body composition and lipid metabolism in rats. *PLoS One* 14:e0210760. 10.1371/journal.pone.0210760 30629707 PMC6328234

[B3] Álvarez-Rendón J. P. Salceda R. Riesgo-Escovar J. R. (2018). *Drosophila melanogaster* as a model for diabetes type 2 progression. *Biomed. Res. Int.* 2018:1417528. 10.1155/2018/1417528 29854726 PMC5941822

[B4] Arruda A. P. Hotamisligil G. S. (2015). Calcium homeostasis and organelle function in the pathogenesis of obesity and diabetes. *Cell Metab.* 22 381–397. 10.1016/j.cmet.2015.06.010 26190652 PMC4558313

[B5] Baker K. D. Thummel C. S. (2007). Diabetic larvae and obese flies-emerging studies of metabolism in *Drosophila*. *Cell Metab.* 6 257–266. 10.1016/j.cmet.2007.09.002 17908555 PMC2231808

[B6] Baumbach J. Hummel P. Bickmeyer I. Kowalczyk K. M. Frank M. Knorr K. (2014a). A *Drosophila in vivo* screen identifies store-operated calcium entry as a key regulator of adiposity. *Cell Metab.* 19 331–343. 10.1016/j.cmet.2013.12.004 24506874

[B7] Baumbach J. Xu Y. Hehlert P. Kühnlein R. P. (2014b). Gαq, Gγ1 and Plc21C control *Drosophila* body fat storage. *J. Genet. Genomics* 41 283–292. 10.1016/j.jgg.2014.03.005 24894355

[B8] Bi J. Wang W. Liu Z. Huang X. Jiang Q. Liu G. (2014). Seipin promotes adipose tissue fat storage through the ER Ca2+-ATPase SERCA. *Cell Metab.* 19 861–871. 10.1016/j.cmet.2014.03.028 24807223

[B9] Birse R. T. Choi J. Reardon K. Rodriguez J. Graham S. Diop S. (2010). High-fat-diet-induced obesity and heart dysfunction are regulated by the TOR pathway in *Drosophila*. *Cell Metab.* 12 533–544. 10.1016/j.cmet.2010.09.014 21035763 PMC3026640

[B10] Buch S. Melcher C. Bauer M. Katzenberger J. Pankratz M. J. (2008). Opposing effects of dietary protein and sugar regulate a transcriptional target of *Drosophila* insulin-like peptide signaling. *Cell Metab.* 7 321–332. 10.1016/j.cmet.2008.02.012 18396138

[B11] Cambron L. D. Thapa G. Greenlee K. J. (2019). Effects of high-fat diet on feeding and performance in the tobacco hornworm, *Manduca sexta*. *Comp. Biochem. Physiol. A Mol. Integr. Physiol.* 236:110526. 10.1016/j.cbpa.2019.110526 31302290 PMC6785999

[B12] Carvalho M. Sampaio J. L. Palm W. Brankatschk M. Eaton S. Shevchenko A. (2012). Effects of diet and development on the *Drosophila* lipidome. *Mol. Syst. Biol.* 8:600. 10.1038/msb.2012.29 22864382 PMC3421444

[B13] Chatterjee N. Perrimon N. (2021). What fuels the fly: energy metabolism in *Drosophila* and its application to the study of obesity and diabetes. *Sci. Adv.* 7:eabg4336. 10.1126/sciadv.abg4336 34108216 PMC8189582

[B14] DiAngelo J. R. Birnbaum M. J. (2009). Regulation of fat cell mass by insulin in *Drosophila melanogaster*. *Mol. Cell. Biol.* 29 6341–6352. 10.1128/MCB.00675-09 19822665 PMC2786867

[B15] Dillen S. Verdonck R. Zels S. Van Wielendaele P. Vanden Broeck J. (2014). Identification of the short neuropeptide F precursor in the desert locust: evidence for an inhibitory role of sNPF in the control of feeding. *Peptides* 53 134–139. 10.1016/j.peptides.2013.09.018 24128610

[B16] Doğan C. Hänniger S. Heckel D. G. Coutu C. Hegedus D. D. Crubaugh L. (2021a). Characterization of calcium signaling proteins from the fat body of the Colorado potato beetle, *Leptinotarsa decemlineata* (Coleoptera: Chrysomelidae): implications for diapause and lipid metabolism. *Insect Biochem. Mol. Biol.* 133:103549. 10.1016/j.ibmb.2021.103549 33610660

[B17] Doğan C. Hänniger S. Heckel D. G. Coutu C. Hegedus D. D. Crubaugh L. (2021b). Two calcium-binding chaperones from the fat body of the Colorado potato beetle, *Leptinotarsa decemlineata* (Coleoptera: Chrysomelidae) involved in diapause. *Arch. Insect Biochem. Physiol.* 106 1–20. 10.1002/arch.21755 33118236

[B18] EPPO (2015). PM 7/124 (1) *Spodoptera littoralis, Spodoptera litura, Spodoptera frugiperda, Spodoptera eridania*. *EPPO Bull.* 45 410–444. 10.1111/epp.12258

[B19] Fernando-Warnakulasuriya G. J. P. Tsuchida K. Wells M. A. (1988). Effect of dietary-lipid content on lipid transport and storage during larval development of *Manduca sexta*. *Insect Biochem.* 18 211–214.

[B20] Friedman S. Waldbauer G. P. Eertmoed J. E. Naeem M. Ghent A. W. (1991). Blood trehalose levels have a role in the control of dietary self-selection by *Heliothis zea* larvae. *J. Insect Physiol.* 37 919–928. 10.1016/0022-1910(91)90007-M

[B21] Gäde G. Auerswald L. (2003). Mode of action of neuropeptides from the adipokinetic hormone family. *Gen. Comp. Endocrinol.* 132 10–20. 10.1016/S0016-6480(03)00159-X 12765639

[B22] Gáliková M. Diesner M. Klepsatel P. Hehlert P. Xu Y. Bickmeyer I. (2015). Energy homeostasis control in *Drosophila* adipokinetic hormone mutants. *Genetics* 201 665–683. 10.1534/genetics.115.178897 26275422 PMC4596676

[B23] Gautam U. K. Hlávková D. Shaik H. A. Karaca I. Karaca G. Sezen K. (2020). Adipokinetic hormones enhance the efficacy of the entomopathogenic fungus *Isaria fumosorosea* in model and pest ıınsects. *Pathogens* 9:801. 10.3390/pathogens9100801 32998278 PMC7600585

[B24] Grönke S. Müller G. Hirsch J. Fellert S. Andreou A. Haase T. (2007). Dual lipolytic control of body fat storage and mobilization in *Drosophila*. *PLoS Biol.* 5:e137. 10.1371/journal.pbio.0050137 17488184 PMC1865564

[B25] Güney G. Toprak U. Hegedus D. D. Bayram Ş Coutu C. Bekkaoui D. (2021). A look into Colorado potato beetle lipid metabolism through the lens of lipid storage droplet proteins. *Insect Biochem. Mol. Biol.* 133:103473. 10.1016/j.ibmb.2020.103473 33010403

[B26] Heinrichsen E. T. Haddad G. G. (2012). Role of high-fat diet in stress response of *Drosophila*. *PLoS One* 7:e0042587. 10.1371/journal.pone.0042587 22870336 PMC3411628

[B27] Heinrichsen E. T. Zhang H. Robinson J. E. Ngo J. Diop S. Bodmer R. (2014). Metabolic and transcriptional response to a high-fat diet in *Drosophila melanogaster*. *Mol. Metab.* 3 42–54. 10.1016/j.molmet.2013.10.003 24567903 PMC3929909

[B28] Hosny M. M. Topper C. P. Moawad G. M. El-Saadany G. B. (1986). Economic damage thresholds of *Spodoptera littoralis* (Boisd.) (Lepidoptera: Noctuidae) on cotton in Egypt. *Crop Prot.* 5 100–104. 10.1016/0261-2194(86)90088-8

[B29] Huang R. Song T. Su H. Lai Z. Qin W. Tian Y. (2020). High-fat diet enhances starvation-induced hyperactivity via sensitizing hunger-sensing neurons in *Drosophila*. *eLife* 9:e53103. 10.7554/eLife.53103 32324135 PMC7274782

[B30] Jacqmain M. Doucet E. Després J. P. Bouchard C. Tremblay A. (2003). Calcium intake, body composition, and lipoprotein-lipid concentrations in adults. *Am. J. Clin. Nutr.* 77 1448–1452. 10.1093/ajcn/77.6.1448 12791622

[B31] Jayakumar S. Richhariya S. Reddy O. V. Texada M. J. Hasan G. (2016). *Drosophila* larval to pupal switch under nutrient stress requires IP3R/Ca(2+) signalling in glutamatergic interneurons. *eLife* 5:e17495. 10.7554/eLife.17495 27494275 PMC4993588

[B32] Jung J. Kim D. I. Han G. Y. Kwon H. W. (2018). The Effects of high fat diet-induced stress on olfactory sensitivity, behaviors, and transcriptional profiling in *Drosophila melanogaster*. *Int. J. Mol. Sci.* 19:2855. 10.3390/ijms19102855 30241362 PMC6213603

[B33] Kim J. Neufeld T. P. (2015). Dietary sugar promotes systemic TOR activation in *Drosophila* through AKH-dependent selective secretion of Dilp3. *Nat. Commun.* 6:6846. 10.1038/ncomms7846 25882208 PMC4402654

[B34] Lee G. Park J. H. (2004). Hemolymph sugar homeostasis and starvation-induced hyperactivity affected by genetic manipulations of the adipokinetic hormone-encoding gene in *Drosophila melanogaster*. *Genetics* 167 311–323. 10.1534/genetics.167.1.311 15166157 PMC1470856

[B35] Lee K. S. You K. H. Choo J. K. Han Y. M. Yu K. (2004). *Drosophila* short neuropeptide F regulates food intake and body size. *J. Biol. Chem.* 279 50781–50789. 10.1074/jbc.M407842200 15385546

[B36] Li Y. Wang S. Liu Y. Lu Y. Zhou M. Wang S. (2020). The Effect of different dietary sugars on the development and fecundity of Harmonia axyridis. *Front. Physiol.* 11:574851. 10.3389/fphys.2020.574851 33041872 PMC7522449

[B37] Liao S. Amcoff M. Nässel D. R. (2021). Impact of high-fat diet on lifespan, metabolism, fecundity and behavioral senescence in *Drosophila*. *Insect Biochem. Mol. Biol.* 133:103495. 10.1016/j.ibmb.2020.103495 33171202

[B38] Lin S. Senapati B. Tsao C. H. (2019). Neural basis of hunger-driven behaviour in *Drosophila*. *Open Biol.* 9:180259. 10.1098/rsob.180259 30914005 PMC6451361

[B39] Matsuda H. Yamada T. Yoshida M. Nishimura T. (2015). Flies without trehalose. *J. Biol. Chem.* 290 1244–1255. 10.1074/jbc.M114.619411 25451929 PMC4294489

[B40] Maus M. Cuk M. Patel B. Lian J. Ouimet M. Kaufmann U. (2017). Store-operated Ca2+ entry controls induction of lipolysis and the transcriptional reprogramming to lipid metabolism. *Cell Metab.* 25 698–712. 10.1016/j.cmet.2016.12.021 28132808 PMC5342942

[B41] May C. E. Rosander J. Gottfried J. Dennis E. Dus M. (2020). Dietary sugar inhibits satiation by decreasing the central processing of sweet taste. *eLife* 9:e54530. 10.7554/eLife.54530 32539934 PMC7297538

[B42] May C. E. Vaziri A. Lin Y. Q. Grushko O. Khabiri M. Wang Q. P. (2019). High dietary sugar reshapes sweet taste to promote feeding behavior in *Drosophila melanogaster*. *Cell Rep.* 27 1675–1685.e7. 10.1016/j.celrep.2019.04.027 31067455 PMC6561488

[B43] Morris S. N. Coogan C. Chamseddin K. Fernandez-Kim S. O. Kolli S. Keller J. N. (2012). Development of diet-induced insulin resistance in adult *Drosophila melanogaster*. *Biochim. Biophys. Acta* 1822 1230–1237. 10.1016/j.bbadis.2012.04.012 22542511 PMC3601833

[B44] Murashov A. K. Pak E. S. Lin C. T. Boykov I. N. Buddo K. A. Mar J. (2020). Preference and detrimental effects of high fat, sugar, and salt diet in wild-caught *Drosophila* simulans are reversed by flight exercise. *FASEB Bioadv.* 3 9–64. 10.1096/fba.2020-00079 33490883 PMC7805546

[B45] Musselman L. P. Fink J. L. Baranski T. J. (2019). Similar effects of high-fructose and high-glucose feeding in a *Drosophila* model of obesity and diabetes. *PLoS One* 14:e0217096. 10.1371/journal.pone.0217096 31091299 PMC6519815

[B46] Musselman L. P. Fink J. L. Narzinski K. Ramachandran P. V. Hathiramani S. S. Cagan R. L. (2011). A high-sugar diet produces obesity and insulin resistance in wild-type *Drosophila*. *Dis. Model Mech.* 4 842–849. 10.1242/dmm.007948 21719444 PMC3209653

[B47] Musselman L. P. Fink J. L. Ramachandran P. V. Patterson B. W. Okunade A. L. Maier E. (2013). Role of fat body lipogenesis in protection against the effects of caloric overload in *Drosophila*. *J. Biol. Chem.* 288 8028–8042. 10.1074/jbc.M112.371047 23355467 PMC3605622

[B48] Musselman L. P. Kühnlein R. P. (2018). *Drosophila* as a model to study obesity and metabolic disease. *J. Exp. Biol.* 221:jeb163881. 10.1242/jeb.163881 29514880

[B49] Na J. Musselman L. P. Pendse J. Baranski T. J. Bodmer R. Ocorr K. (2013). A *Drosophila* model of high sugar diet-induced cardiomyopathy. *PLoS Genet.* 9:e1003175. 10.1371/journal.pgen.1003175 23326243 PMC3542070

[B50] Nagata S. Morooka N. Matsumoto S. Kawai T. Nagasawa H. (2011). Effects of neuropeptides on feeding initiation in larvae of the silkworm, Bombyx mori *Gen. Comp. Endocrinol.* 172 90–95. 10.1016/j.ygcen.2011.03.004 21397600

[B51] Oh Y. Lai J. S. Y. Mills H. J. Erdjument-Bromage H. Giammarinaro B. Saadipour K. (2019). A glucose-sensing neuron pair regulates insulin and glucagon in *Drosophila*. *Nature* 574 559–564. 10.1038/s41586-019-1675-4 31645735 PMC6857815

[B52] Owusu-Ansah E. Perrimon N. (2014). Modeling metabolic homeostasis and nutrient sensing in *Drosophila*: implications for aging and metabolic diseases. *Dis. Model Mech.* 7 343–350. 10.1242/dmm.012989 24609035 PMC3944494

[B53] Palanker Musselman L. Fink J. L. Baranski T. J. (2016). CoA protects against the deleterious effects of caloric overload in *Drosophila*. *J. Lipid Res.* 57 380–387. 10.1194/jlr.M062976 26805007 PMC4766987

[B54] Pasco M. Y. Léopold P. (2012). High sugar-induced insulin resistance in *Drosophila* relies on the lipocalin Neural Lazarillo. *PLoS One* 7:e36583. 10.1371/journal.pone.0036583 22567167 PMC3342234

[B55] Pimentel A. C. Montali A. Bruno D. Tettamanti G. (2017). Metabolic adjustment of the larval fat body in Hermetia illucens to dietary conditions. *J. Asia Pac. Entomol.* 20 1307–1313. 10.1016/j.aspen.2017.09.017

[B56] Piper M. D. Bartke A. (2008). Diet and aging. *Cell Metab.* 8 99–104. 10.1016/j.cmet.2008.06.012 18680711

[B57] Post S. Liao S. Yamamoto R. Veenstra J. A. Nässel D. R. Tatar M. (2019). *Drosophila* insulin-like peptide dilp1 increases lifespan and glucagon-like Akh expression epistatic to dilp2. *Aging Cell* 18:e12863. 10.1111/acel.12863 30511458 PMC6351851

[B58] Rani L. Saini S. Shukla N. Chowdhuri D. K. Gautam N. K. (2020). High sucrose diet induces morphological, structural and functional impairments in the renal tubules of *Drosophila melanogaster*: a model for studying type-2 diabetes mediated renal tubular dysfunction. *Insect Biochem. Mol. Biol.* 125:103441. 10.1016/j.ibmb.2020.103441 32735915

[B59] Reed L. K. Williams S. Springston M. Brown J. Freeman K. DesRochesa C. E. (2010). Genotype-by-diet interactions drive metabolic phenotype variation in *Drosophila melanogaster*. *Genetics* 185 1009–1019. 10.1534/genetics.109.113571 20385784 PMC2907188

[B60] Reiter L. T. Potocki L. Chien S. Gribskov M. Bier E. (2001). A systematic analysis of human disease-associated gene sequences in *Drosophila melanogaster*. *Genome Res.* 11 1114–1125. 10.1101/gr.169101 11381037 PMC311089

[B61] Rivera O. McHan L. Konadu B. Patel S. Sint Jago S. Talbert M. E. (2019). A high-fat diet impacts memory and gene expression of the head in mated female *Drosophila melanogaster*. *J. Comp. Physiol B.* 189 179–198. 10.1007/s00360-019-01209-9 30810797 PMC6711602

[B62] Rovenko B. M. Kubrak O. I. Gospodaryov D. V. Perkhulyn N. V. Yurkevych I. S. Sanz A. (2015a). High sucrose consumption promotes obesity whereas its low consumption induces oxidative stress in *Drosophila melanogaster*. *J. Insect Physiol.* 79 42–54. 10.1016/j.jinsphys.2015.05.007 26050918

[B63] Rovenko B. M. Perkhulyn N. V. Gospodaryov D. V. Sanz A. Lushchak O. V. Lushchak V. I. (2015b). High consumption of fructose rather than glucose promotes a diet-induced obese phenotype in *Drosophila melanogaster*. *Comp. Biochem. Physiol. A Mol. Integr. Physiol.* 180 75–85. 10.1016/j.cbpa.2014.11.008 25461489

[B64] Schultzhaus J. N. Bennett C. J. Iftikhar H. Yew J. Y. Mallett J. Carney G. E. (2018). High fat diet alters *Drosophila melanogaster* sexual behavior and traits: decreased attractiveness and changes in pheromone profiles. *Sci. Rep.* 8:5387. 10.1038/s41598-018-23662-2 29599496 PMC5876352

[B65] Semaniuk U. V. Gospodaryov D. V. Feden’ko K. M. Yurkevych I. S. Vaiserman A. M. Storey K. B. (2018). Insulin-like peptides regulate feeding preference and metabolism in *Drosophila*. *Front. Physiol.* 9:1083. 10.3389/fphys.2018.01083 30197596 PMC6118219

[B66] Shi H. Dirienzo D. Zemel M. B. (2001). Effects of dietary calcium on adipocyte lipid metabolism and body weight regulation in energy-restricted aP2-agouti transgenic mice. *FASEB J.* 15 291–293. 10.1096/fj.00-0584fje 11156940

[B67] Slade J. D. Staveley B. E. (2016). Manipulation of components that control feeding behavior in *Drosophila melanogaster* increases sensitivity to amino acid starvation. *Genet. Mol. Res.* 15 1–12. 10.4238/gmr.15017489 26909968

[B68] Song W. Cheng D. Hong S. Sappe B. Hu Y. Wei N. (2017). Midgut-derived activin regulates glucagon-like action in the fat body and glycemic control. *Cell Metab.* 25 386–399. 10.1016/j.cmet.2017.01.002 28178568 PMC5373560

[B69] Staats S. Lüersen K. Wagner A. E. Rimbach G. (2018). *Drosophila melanogaster* as a versatile model organism in food and nutrition research. *J. Agric. Food Chem.* 66 3737–3753. 10.1021/acs.jafc.7b05900 29619822

[B70] Stobdan T. Sahoo D. Azad P. Hartley I. Heinrichsen E. Zhou D. (2019). High fat diet induces sex-specific differential gene expression in *Drosophila melanogaster*. *PLoS One* 14:e0213474. 10.1371/journal.pone.0213474 30861021 PMC6413938

[B71] Subramanian M. Jayakumar S. Richhariya S. Hasan G. (2013a). Loss of IP3 receptor function in neuropeptide secreting neurons leads to obesity in adult *Drosophila*. *BMC Neurosci.* 14:157. 10.1186/1471-2202-14-157 24350669 PMC3878400

[B72] Subramanian M. Metya S. K. Sadaf S. Kumar S. Schwudke D. Hasan G. (2013b). Altered lipid homeostasis in *Drosophila* InsP3 receptor mutants leads to obesity and hyperphagia. *Dis. Model Mech.* 6 734–744. 10.1242/dmm.010017 23471909 PMC3634656

[B73] Sun X. Zemel M. B. (2004). Calcium and dairy products inhibit weight and fat regain during ad libitum consumption following energy restriction in Ap2-agouti transgenic mice. *J. Nutr.* 134 3054–3060. 10.1093/jn/134.11.3054 15514275

[B74] Tatum E. L. Beadle G. W. (1939). Effect of diet on eye-color development in *Drosophila melanogaster*. *Biol. Bull.* 77 415–422. 10.2307/1537651

[B75] Thompson S. N. Borchardt D. B. Wang L. W. (2003). Dietary nutrient levels regulate protein and carbohydrate intake, gluconeogenic/glycolytic flux and blood trehalose level in the insect *Manduca sexta* L. *J. Comp. Physiol. B* 173 149–163. 10.1007/s00360-002-0322-8 12624653

[B76] Toprak U. (2020). The role of peptide hormones in insect lipid metabolism. *Front. Physiol.* 11:434. 10.3389/fphys.2020.00434 32457651 PMC7221030

[B77] Toprak U. Bayram S. Gürkan O. M. (2006). Comparative biological activities of a plaque-purified variant and a Turkish native isolate of SpliNPV-B against *Spodoptera littoralis* (Lepidoptera: Noctuidae). *Pest Manag. Sci.* 62 57–63. 10.1002/ps.1128 16235266

[B78] Toprak U. Doğan C. Hegedus D. (2021). A comparative perspective on functionally-related, intracellular calcium channels: the insect ryanodine and inositol 1,4,5-trisphosphate receptors. *Biomolecules* 11:1031. 10.3390/biom11071031 34356655 PMC8301844

[B79] Toprak U. Güz N. Gürkan M. O. Hegedus D. D. (2014). Identification and coordinated expression of perilipin genes in the biological cycle of sunn pest, *Eurygaster maura* (Hemiptera: Scutelleridae): implications for lipolysis and lipogenesis. *Comp. Biochem. Physiol. B Biochem. Mol. Biol.* 171 1–11. 10.1016/j.cbpb.2014.02.001 24556114

[B80] Toprak U. Hegedus D. Doğan C. Güney G. (2020). A journey into the world of insect lipid metabolism. *Arch. Insect Biochem. Physiol.* 104 1–67. 10.1002/arch.21682 32335968

[B81] Trindade de Paula M. Poetini Silva M. R. Machado Araujo S. Cardoso Bortolotto V. Barreto Meichtry L. Zemolin A. P. (2016). High-fat diet ıınduces oxidative stress and mpk2 and hsp83 gene expression in *Drosophila melanogaster*. *Oxid. Med. Cell. Longev.* 2016:4018157. 10.1155/2016/4018157 27579152 PMC4992541

[B82] Ugrankar R. Berglund E. Akdemir F. Tran C. Kim M. S. Noh J. (2015). *Drosophila* glucome screening identifies Ck1alpha as a regulator of mammalian glucose metabolism. *Nat. Commun.* 6:7102. 10.1038/ncomms8102 25994086 PMC4455130

[B83] Van de Velde S. Badisco L. Claeys I. Verleyen P. Chen X. Vanden Bosch L. (2007). Insulin-like peptides in *Spodoptera littoralis* (Lepidoptera): detection, localization and identification. *Gen. Comp. Endocrinol.* 153 72–79. 10.1016/j.ygcen.2007.05.001 17559850

[B84] van Schoor T. Kelly E. T. Tam N. Attardo G. M. (2020). Impacts of dietary nutritional composition on larval development and adult body composition in the yellow fever mosquito (Aedes aegypti). *Insects* 11:535. 10.3390/insects11080535 32824225 PMC7469193

[B85] Večeřa J. Krishnan N. Mithöfer A. Vogel H. Kodrík D. (2012). Adipokinetic hormone-induced antioxidant response in *Spodoptera littoralis*. *Comp. Biochem. Physiol. C Toxicol. Pharmacol.* 155 389–395. 10.1016/j.cbpc.2011.10.009 22085825

[B86] Woodcock K. J. Kierdorf K. Pouchelon C. A. Vivancos V. Dionne M. S. Geissmann F. (2015). Macrophage-derived upd3 cytokine causes impaired glucose homeostasis and reduced lifespan in *Drosophila* fed a lipid-rich diet. *Immunity* 42 133–144. 10.1016/j.immuni.2014.12.023 25601202 PMC4304720

[B87] Wu Q. Wen T. Lee G. Park J. H. Cai H. N. Shen P. (2003). Developmental control of foraging and social behavior by the *Drosophila* neuropeptide Y-like system. *Neuron* 39 147–161. 10.1016/s0896-6273(03)00396-9 12848939

[B88] Yasugi T. Yamada T. Nishimura T. (2017). Adaptation to dietary conditions by trehalose metabolism in *Drosophila*. *Sci. Rep.* 7:1619. 10.1038/s41598-017-01754-9 28487555 PMC5431645

[B89] Zanotto F. P. Raubenheimer D. Simpson S. J. (1996). Haemolymph amino acid and sugar levels in locusts fed nutritionally unbalanced diets. *J. Comp. Physiol. B* 166 223–229. 10.1007/BF00263986

[B90] Zemel M. B. (2002). Regulation of adiposity and obesity risk by dietary calcium: mechanisms and implications. *J. Am. Coll. Nutr.* 21 146S–151S. 10.1080/07315724.2002.10719212 11999543

[B91] Zemel M. B. Shi H. Greer B. Dirienzo D. Zemel P. C. (2000). Regulation of adiposity by dietary calcium. *FASEB J.* 14 1132–1138. 10.1096/fasebj.14.9.1132 10834935

[B92] Zinke I. Schütz C. S. Katzenberger J. D. Bauer M. Pankratz M. J. (2002). Nutrient control of gene expression in *Drosophila*: microarray analysis of starvation and sugar-dependent response. *EMBO J.* 21 6162–6173. 10.1093/emboj/cdf600 12426388 PMC137192

